# Performance of Fully Automated Plasma Assays as Screening Tests for Alzheimer Disease–Related β-Amyloid Status

**DOI:** 10.1001/jamaneurol.2019.1632

**Published:** 2019-06-24

**Authors:** Sebastian Palmqvist, Shorena Janelidze, Erik Stomrud, Henrik Zetterberg, Johann Karl, Katharina Zink, Tobias Bittner, Niklas Mattsson, Udo Eichenlaub, Kaj Blennow, Oskar Hansson

**Affiliations:** 1 Clinical Memory Research Unit, Department of Clinical Sciences, Lund University, Lund, Sweden; 2 Department of Neurology, Skåne University Hospital, Malmö, Sweden; 3 Memory Clinic, Skåne University Hospital, Malmö, Sweden; 4 Department of Psychiatry and Neurochemistry, the Sahlgrenska Academy at the University of Gothenburg, Mölndal, Sweden; 5 Clinical Neurochemistry Laboratory, Sahlgrenska University Hospital, Mölndal, Sweden; 6 Department of Neurodegenerative Disease, UCL Institute of Neurology, Queen Square, London, United Kingdom; 7 UK Dementia Research Institute at UCL, London, United Kingdom; 8 Roche Diagnostics GmbH, Penzberg, Germany; 9 Genentech, a Member of the Roche Group, Basel, Switzerland

## Abstract

**Importance:**

Accurate blood-based biomarkers for Alzheimer disease (AD) might improve the diagnostic accuracy in primary care, referrals to memory clinics, and screenings for AD trials.

**Objective:**

To examine the accuracy of plasma β-amyloid (Aβ) and tau measured using fully automated assays together with other blood-based biomarkers to detect cerebral Aβ.

**Design, Setting, and Participants:**

Two prospective, cross-sectional, multicenter studies. Study participants were consecutively enrolled between July 6, 2009, and February 11, 2015 (cohort 1), and between January 29, 2000, and October 11, 2006 (cohort 2). Data were analyzed in 2018. The first cohort comprised 842 participants (513 cognitively unimpaired [CU], 265 with mild cognitive impairment [MCI], and 64 with AD dementia) from the Swedish BioFINDER study. The validation cohort comprised 237 participants (34 CU, 109 MCI, and 94 AD dementia) from a German biomarker study.

**Main Outcome and Measures:**

The cerebrospinal fluid (CSF) Aβ42/Aβ40 ratio was used as the reference standard for brain Aβ status. Plasma Aβ42, Aβ40 and tau were measured using Elecsys immunoassays (Roche Diagnostics) and examined as predictors of Aβ status in logistic regression models in cohort 1 and replicated in cohort 2. Plasma neurofilament light chain (NFL) and heavy chain (NFH) and *APOE* genotype were also examined in cohort 1.

**Results:**

The mean (SD) age of the 842 participants in cohort 1 was 72 (5.6) years, with a range of 59 to 88 years, and 446 (52.5%) were female. For the 237 in cohort 2, mean (SD) age was 66 (10) years with a range of 23 to 85 years, and 120 (50.6%) were female. In cohort 1, plasma Aβ42 and Aβ40 predicted Aβ status with an area under the receiver operating characteristic curve (AUC) of 0.80 (95% CI, 0.77-0.83). When adding *APOE*, the AUC increased significantly to 0.85 (95% CI, 0.82-0.88). Slight improvements were seen when adding plasma tau (AUC, 0.86; 95% CI, 0.83-0.88) or tau and NFL (AUC, 0.87; 95% CI, 0.84-0.89) to Aβ42, Aβ40 and *APOE*. The results were similar in CU and cognitively impaired participants, and in younger and older participants. Applying the plasma Aβ42 and Aβ40 model from cohort 1 in cohort 2 resulted in slightly higher AUC (0.86; 95% CI, 0.81-0.91), but plasma tau did not contribute. Using plasma Aβ42, Aβ40, and *APOE* in an AD trial screening scenario reduced positron emission tomography costs up to 30% to 50% depending on cutoff.

**Conclusions and Relevance:**

Plasma Aβ42 and Aβ40 measured using Elecsys immunoassays predict Aβ status in all stages of AD with similar accuracy in a validation cohort. Their accuracy can be further increased by analyzing *APOE* genotype. Potential future applications of these blood tests include prescreening of Aβ positivity in clinical AD trials to lower the costs and number of positron emission tomography scans or lumbar punctures.

## Key Points

**Question:**

Do plasma levels of β-amyloid 42, β-amyloid 40, and tau detect cerebral β-amyloid status when measured using fully automated immunoassays?

**Findings:**

In 2 cross-sectional studies, plasma β-amyloid 42 to β-amyloid 40 ratio, measured using immunoassay, accurately predicted cerebral β-amyloid status in all stages of Alzheimer disease in the BioFINDER cohort (n = 842) and in an independent validation cohort (n = 237). The diagnostic accuracy was further increased by analyzing *APOE* genotype.

**Meaning:**

Blood-based β-amyloid 42 and β-amyloid 40 ratio together with *APOE* genotype may be used as prescreening tests in primary care and in clinical Alzheimer disease trials to lower the costs and number of positron emission tomography scans and lumbar punctures.

## Teaser

This corss-sectional diagnostic study evaluates the accuracy of fully automated plasma assays in measuring plasma β-amyloid and tau in patients with and without cognitive impairment in the Swedish BioFINDER study and an independent validation cohort.

## Introduction

A key hallmark of Alzheimer disease (AD) is the gradual accumulation of β-amyloid (Aβ) in the brain, which starts decades before the onset of cognitive symptoms. Detection of abnormal Aβ accumulation (Aβ positivity) may support the clinical diagnosis of AD^1,2^ and is essential for including participants in clinical AD trials targeting Aβ.^3^ β-Amyloid can be detected in vivo using positron emission tomography (PET) with ligands that bind to Aβ fibrils or by measuring the levels of the peptide Aβ1-42 (Aβ42) in cerebrospinal fluid (CSF).^4^ Alzheimer disease affects 1 in 10 persons aged 65 years and older and is expected to affect more than 100 million people by 2050.^5,6^ The costs and limited access to PET or CSF analysis may restrict their use to a minority of cases. There is thus a great need for readily available methods that can detect brain Aβ, and perhaps the most desirable goal has been to establish blood-based biomarkers of Aβ. Many candidate blood biomarkers have failed in replication studies,^7,8^ but somewhat promising results have been seen for plasma tau, neurofilament light chain (NFL), and combinations of Aβ42 and Aβ40.^9,10,11,12,13,14,15,16,17^ Although there are diagnostic inconsistencies regarding the plasma Aβ42/Aβ40 ratio in older studies,^18^ more recent studies have demonstrated that it correlates with brain Aβ and can differentiate patients with AD from healthy control participants.^12,13^ Most recently, 2 independent groups demonstrated improved accuracy for plasma Aβ42/Aβ40 using immunoprecipitation–mass spectrometry assays.^19,20^ Although these studies are promising and show the potential of plasma Aβ as a true AD biomarker, they are costly and need extensive development before they can be implemented in primary care or in large screenings where cost-effective, fully automated, high-throughput, and highly reliable analysis methods are needed.

Measuring plasma Aβ presents the same challenges as measuring CSF Aβ in that several analysis methods exist and unified cutoffs have been difficult to establish, even using the same assay, owing to high variability between laboratories and assay batches.^21,22^ Recently, fully automated immunoassays have been developed by several different vendors with improved reliability and precision for CSF Aβ and tau species.^23,24,25^ For example, for the Elecsys immunoassays (Roche Diagnostics), it has been shown that CSF cutoffs established in one European cohort could be applied to another independent cohort in the United States to determine amyloid PET status with high accuracy.^24^

Using these newly developed Elecsys assays for detection of Aβ42, Aβ40, and tau, our aims were to examine the accuracy of plasma Aβ42, Aβ40, and tau to estimate Aβ positivity, whether the accuracy could be improved by adding plasma neurofilament (light and heavy chain) and *APOE* genotype to the models, and how the Elecsys assays perform in an independent validation cohort.

## Methods

### Participants

The study population was included from the prospective Swedish BioFINDER Study, which enrolled participants between July 6, 2009, to February 11, 2015, from the southern part of Sweden. Of all 892 participants in BioFINDER’s control, mild cognitive symptoms, and AD cohorts, plasma samples were hemolyzed or not available in sufficient amount for 50 individuals. Thus, 842 participants could be included in the present study. They were classified as cognitively unimpaired^26^ (CU; 513 participants, of whom 195 had subjective cognitive decline)^27^; mild cognitive impairment^28^ (MCI; 265 participants); or AD dementia^2^ (64 participants). In subsample analyses, we grouped the population into CU and cognitively impaired (MCI + AD), because all participants with AD were Aβ positive and therefore could not be examined separately using Aβ status as outcome. Study design and specific inclusion and exclusion criteria are described elsewhere^29^ (eMethods in the Supplement). The study was approved by the Regional Ethics Committee in Lund, Sweden, and all participants gave their written informed consent to participate in the study. For the independent validation cohort, the study was approved by the ethical committee of the Medizinische Hochschule in Hannover and the ethical committee of the University of Ulm, and all participants gave written informed consent.

### Plasma and CSF Procedures

Blood samples were collected at the same time as CSF samples, and the collection was performed in the morning with participants not fasting. Blood samples were collected and analyzed according to a standardized protocol. For each study participant, blood was collected in 6 EDTA-plasma tubes (Vacutainer K_2_EDTA tube; BD Diagnostics) and centrifuged (2000 *g*, 4°C) for 10 minutes. After centrifugation, plasma from all 6 tubes was transferred into one 50-mL tube (62.547.254, Sarstedt), mixed, and 1 mL was aliquoted into polypropylene tubes (72.694.100; Sarstedt) and stored at –80°C within 30 to 60 minutes of collection. All plasma samples went through 1 freeze-thaw cycle before the analysis, when 300 μL was further aliquoted into Lobind tubes (72.704.600; Sarstedt). The current standardized protocol is consistent with recent findings that blood must be centrifuged within 1 hour and frozen shortly thereafter; however, up to 3 freeze-thaw cycles and 5 tube transfers do not affect plasma Aβ and tau values.^30^ Lumbar puncture and CSF handling followed a structured protocol.^31^ Plasma and CSF Aβ42, Aβ40, total tau (tau), and phosphorylated tau (P-tau; only in CSF) were analyzed using the Elecsys immunoassays on a cobas e 601 analyzer (Roche Diagnostics) at the Clinical Neurochemistry Laboratory, University of Gothenburg, Sweden. Additional assay data (also including NFL and neurofilament heavy chain [NFH] analyses) can be found in the eMethods and eTables 1 and 2 in the Supplement.

### Reference Standard for Aβ Status

β-Amyloid status was determined using the Elecsys CSF Aβ42/Aβ40 ratio, which is a ratio that has been validated against amyloid PET status with more than 90% agreement.^32,33,34^ An unbiased cutoff of less than 0.059 was used to define Aβ positivity based on mixture modeling statistics, which previously has proved to provide robust and accurate thresholds.^35,36^ In a secondary analysis (eFigure 3 and eFigure 4 in the Supplement), we used the Elecsys CSF P-tau/Aβ42 ratio to define Aβ positivity, using the predefined cutoff of 0.022 or greater.^24^

### Independent Validation Cohort

All 237 participants of this study were enrolled between January 29, 2000, and October 11, 2006, at 2 clinical sites in Germany, Ulm and Hannover, as part of a prospective validation study of new biomarkers for the early diagnosis of AD. The participants were classified as having CU (n = 34), MCI (n = 109),^37^ or AD mild dementia^38^ (Mini-Mental State Examination score >22; n = 94). Specific inclusion/exclusion criteria and CSF and blood collection procedures^30^ are described in the eMethods in the Supplement. The cutoff of CSF Aβ42/Aβ40 of less than 0.059 established in BioFINDER to define Aβ positivity was also used in the validation cohort after a thorough assessment of the CSF Aβ42/Aβ40 distribution. As in BioFINDER, the previously published cutoff of CSF P-tau/Aβ42 0.022 or greater^24^ was used as a secondary reference standard for Aβ status also in the validation cohort.

### Statistical Analysis

According to previous publications^39,40^ and present analyses (eResults in the Supplement), *APOE* (OMIM:107741) genotype analyzed from blood was grouped into (A) ε2/ε2 or ε2/ε3; (B) ε3/ε3; (C) ε2/ε4 or ε3/ε4; and (D) ε4/ε4. *APOE* ε3/ε3 was the reference category in the statistical models. β-Amyloid status was predicted in logistic regression models to produce estimates of the predictors, probabilities for Aβ positivity, and resulting area under the receiver operating characteristic curve (AUC). The examined predictors in BioFINDER were the plasma biomarkers Aβ42, Aβ40, tau, NFH, and NFL and *APOE* genotype. The models were built using the Akaike information criterion (AIC) to evaluate the model fit. A predictor was kept in the model if AIC improved significantly (a decrease in AIC of at least 2, noted as “ΔAIC -2”).^41^ Differences in AUCs were compared using DeLong statistics.^42^ In the replication analysis, the models (intercepts and estimates) established in BioFINDER were applied to the validation cohort. The resulting probabilities from the validation cohort were used to calculate the AUCs (only plasma Aβ42, Aβ40, and tau were available in this cohort). Additional statistical methods are described in the eMethods in the Supplement. SPSS version 24 (IBM) and R version 3.4 (R Foundation for Statistical Computing) were used for the statistical analyses. Two-sided *P* < .05 indicated statistical significance.

## Results

Among the 842 study participants in BioFINDER, mean (SD) age was 72.0 (5.6) years, and 446 (52.5%) were female. Demographic and clinical data for the study participants in BioFINDER are shown in Table 1. In the total BioFINDER population of 842, 368 were positive for Aβ (prevalence, 44%); 147 of 513 with CU (29%) were positive; 157 of 265 (60%) with MCI; and, by definition, all 64 (100%) with AD dementia.

**Table 1.** Demographic and Clinical Data^a^

Characteristic	CU Aβ− (n = 366)	CU Aβ+ (n = 147)	MCI Aβ− (n = 108)	MCI Aβ+ (n = 157)	AD Aβ+ (n = 64)
Sex					
Male	152	69	76^b^	79	25
Female	214	78	32	78	39
Age, y	72 (5)	73 (5)	69 (6)^b^	72 (5)	76 (5)^b^
*APOE* genotype, %					
1 or 2 ε4 alleles,	19	63^b^	24	70^b^	69^b^
MMSE	28.9 (1.1)	28.6 (1.3)	27.5 (1.8)^b^	26.7 (1.8)^b^	21.8 (3.7)^b^
Delayed recall (ADAS-cog; errors)^c^	2.2 (1.9)	3.2 (2.3)^b^	5.7 (2.4)^b^	7.0 (2.1)^b^	8.6 (1.6)^b^
**CSF**					
Aβ42, pg/mL	1665 (596)	819 (303)^b^	1572 (605)	706 (256)^b^	671 (315)^b^
Aβ40, ng/mL	18.2 (5.2)	19.5 (5.9)^d^	17.3 (5.7)	17.8 (5.0)	17.9 (6.2)
Aβ42/Aβ40	0.091 (0.016)	0.042 (0.009)^b^	0.090 (0.014)	0.040 (0.098)^b^	0.037 (0.009)^b^
T-tau, pg/mL	209 (62)	309 (112)^b^	209 (76)	341 (136)^b^	384 (143)^b^
P-tau, pg/mL	17.5 (5.3)	28.5 (12.0)^b^	16.9 (6.4)	32.2 (14.5) ^b^	36.3 (16.3)^e^
NFL, pg/mL	918 (490)	1216 (842)^b^	1648 (1517)^b^	1531 (1195)^b^	2002 (1835)^b^
NFH, pg/mL	504 (190)	584 (241)^b^	641 (463)^b^	637 (303) ^b^	821 (687)^b^
**Plasma**					
Aβ42, pg/mL	32.8 (4.9)	29.6 (4.3)^b^	33.1 (5.2)	30.3 (4.5)^b^	23.3 (8.2)^b^
Aβ40, pg/mL	482 (63.3)	479 (67.5)	495 (83.2)	492 (75.4)	380 (131.7)^e^
T-tau, pg/mL	16.6 (4.7)	17.9 (5.4)^e^	18.7 (6.1)^b^	19.1 (5.2)^b^	16.7 (6.0)
Aβ42/Aβ40	0.068 (0.007)	0.062 (0.007)^b^	0.067 (0.007)	0.062 (0.006)^b^	0.062 (0.010)^b^
NFL, pg/mL	21.0 (11.8)	29.1 (59.6)^e^	28.3 (28.4)^b^	29.0 (17.9)^b^	43.8 (28.7)^b^
NFH, pg/mL^f^	51.4 (68.2)	53.7 (48.7)	59.7 (55.1)	65.9 (56.6)^b^	79.8.4 (77.0)^b^

Abbreviations: Aβ, β-amyloid; Aβ+, Aβ positive; Aβ−, Aβ negative; CSF, cerebrospinal fluid; MMSE, Mini-Mental State Examination; NHL, neurofilament heavy chain; NFL, neurofilament light chain.

^a^ β-Amyloid status was defined based on a CSF Aβ42/Aβ40 cutoff of ≤0.059. Data are shown as mean (SD) unless otherwise specified. Demographic factors, clinical characteristics, and biomarkers levels were compared using χ^2^ test and 1-way analysis of variance (not adjusted for multiple comparisons). Neurofilament light chain and NFH values were ln-transformed before the analysis. In the receiver operating characteristic subanalyses, the mild cognitive impairment and Alzheimer disease cohorts are combined as cognitively impaired (Figure 3A and B; eFigure 3C and D; eTable 4 in the Supplement). When calculating the Aβ42/Aβ40 ratio, picomolar per milliliter was used for both peptides.

^b^ *P* < .001 compared with CU Aβ−.

^c^ Data were missing for 1 CU Aβ−, 1 MCI Aβ−, 8 MCI Aβ+ and 5 AD Aβ+ individuals.

^d^ *P* < .05.

^e^ *P* < .01.

^f^ Data were missing for 6 CU Aβ−, 3 CU Aβ+, 2 MCI Aβ−, 5 MCI Aβ+ and 5 AD Aβ+ individuals.

### Correlations Between Plasma and CSF Biomarkers

In the whole BioFINDER population, there were statistically significant positive correlations between all plasma and corresponding CSF biomarkers (eTable 3 in the Supplement). The correlations were similar within diagnostic subgroups (eFigure 1 and eTable 3 in the Supplement).

### Plasma Aβ and tau Levels in Diagnostic Groups

**Figure 1.**
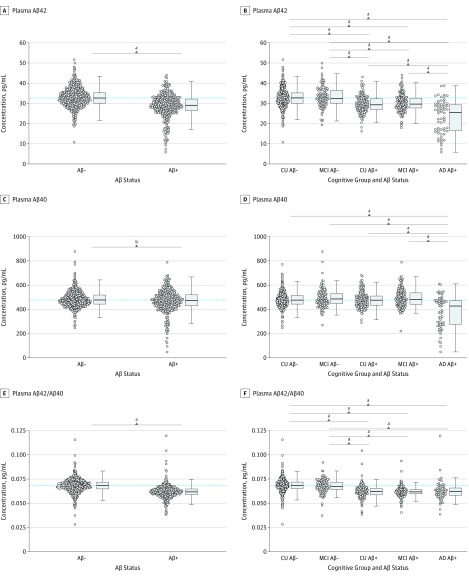
Levels of Plasma β-Amyloid (Aβ) Biomarkers Plasma Aβ42 (A), Aβ40 (C), and the plasma Aβ42/Aβ40 ratio (E) in the Aβ-positive (Aβ+) (CSF Aβ42/Aβ40 ≤ 0.059) and Aβ-negative (Aβ−) (CSF Aβ42/Aβ40 > 0.059) groups. Plasma Aβ42 (B), Aβ40 (D), and the plasma Aβ42/Aβ40 ratio (F) in the CU, MCI, and AD participant groups stratified by Aβ status. The dotted lines indicate median levels in the CU Aβ-negative group. *P* values are calculated from *t* test (A, C, E) or 1-way analysis of variance and post hoc tests with the statistical significance set to *P* < .005 (.05/10.00) to account for the Bonferroni correction (B, D, F). The significant findings were similar when adjusting for age and sex (data not shown). Group comparisons of plasma tau, NFH, and NFL are shown in eFigure 2 in the Supplement. AD, Alzheimer disease; CSF, cerebrospinal fluid; CU, cognitively unimpaired; MCI, mild cognitive impairment; NFH, neurofilament heavy chain; and NFL, neurofilament light chain.

In BioFINDER, plasma levels of Aβ42, Aβ40, and Aβ42/Aβ40 were decreased in Aβ-positive (CSF Aβ42/Aβ40 ≤ 0.059) compared with Aβ-negative (CSF Aβ42/Aβ40 > 0.059) participants (Aβ42, *P* < .001; Aβ40 *P* = .003, Aβ42/Aβ42, *P* < .001; Figure 1A-C). When comparing Aβ groups stratified by diagnostic subgroup, plasma levels of Aβ42 were lower in the CU Aβ-positive, MCI Aβ-positive and AD Aβ-positive dementia groups compared with the CU Aβ-negative and MCI Aβ-negative groups (*P* < .001 for all; Figure 1D). The decrease in plasma Aβ42 was more pronounced in AD Aβ-positive dementia compared with CU Aβ-positive and MCI Aβ positive groups. Plasma Aβ40 levels were lower in the AD Aβ-positive dementia group compared with all other groups (*P* < .001 for all), but there were no differences between CU Aβ-negative, CU Aβ-positive, and MCI Aβ-positive participants (Figure 1E). The plasma Aβ42/Aβ40 ratio was lower in the CU Aβ-positive, MCI Aβ-positive, and AD Aβ-positive dementia groups than in the CU Aβ-negative and MCI Aβ-negative groups with no differences across the Aβ-positive groups (Figure 1F). The significant findings were very similar when adjusting for age and sex (data not shown). Comparisons of plasma tau, NFL, and NFH are shown in eFigure 2 in the Supplement.

### Accuracy of Plasma Aβ42 and Aβ40 for Predicting Brain Aβ Positivity

**Figure 2.**
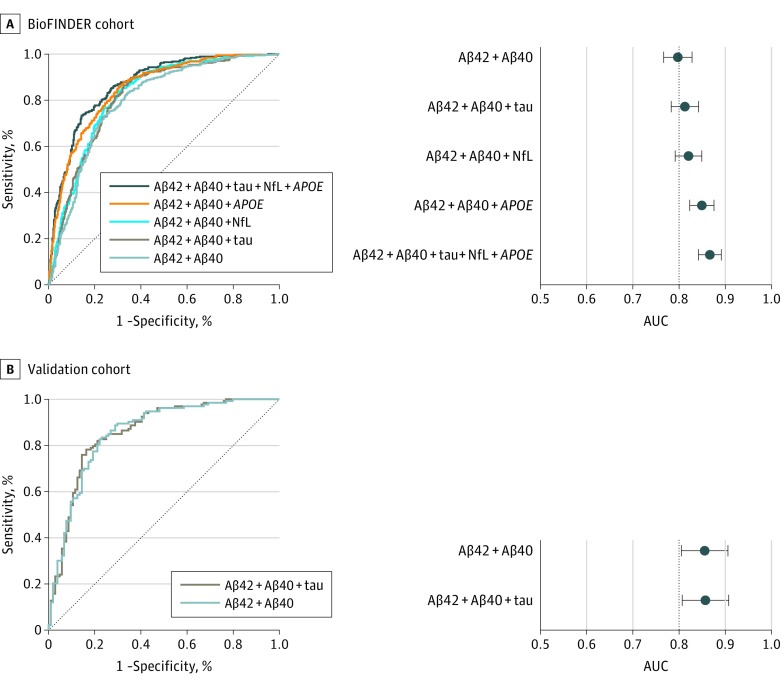
Receiver Operating Characteristic (ROC) Analysis of Plasma Biomarkers in the BioFINDER and Validation Cohorts Optimized ROC curves and corresponding areas under the curve (AUCs) for plasma Aβ together with the additional predictors, *APOE*, plasma tau, and neurofilament light chain (NFL) to assess accuracy when predicting Aβ positivity (crebrospinal fluid Aβ42/Aβ40 ≤ 0.059) in the BioFINDER (A and B, n = 842); and the replication of these models (C and D, n = 237) in the validation cohort using the estimates and intercepts established in BioFINDER. *APOE* genotype and NFL were not available in the validation cohort. Error bars indicate 95% CIs. ROC analyses in subpopulations can be found in Figure 3 and eTable 4 and 6 in the Supplement. Sensitivities and specificities are shown in Table 2. ROC analyses using the alternative reference standard for Aβ positivity (CSF P-tau/Aβ42 ≥ 0.022) are shown in eFigures 3 and 4 in the Supplement.

**Figure 3.**
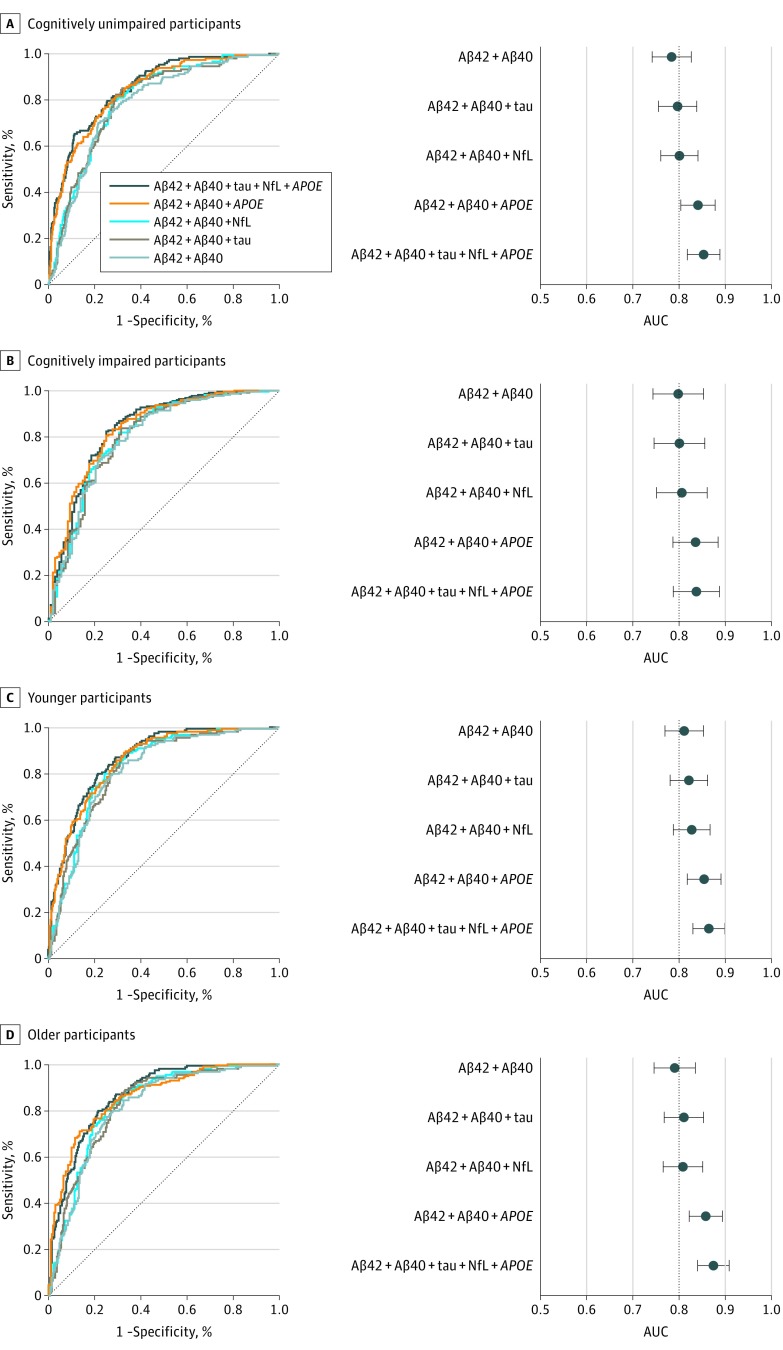
Receiver Operating Characteristic (ROC) Analysis of Plasma Biomarkers in Subpopulations in BioFINDER ROC curves and corresponding areas under the curve (AUCs) from logistic regression models for plasma Aβ together with the additional predictors *APOE*, plasma tau, and neurofilament light chain (NFL), to assess accuracy when detecting Aβ positivity (cerebrospinal fluid Aβ42/Aβ40 ≤ 0.059) in cognitively unimpaired participants (A and B, n = 513), cognitively impaired participants (C and D, n = 329), the younger half of the cohort (E and F, n = 428; 60-72 y), and the older half of the cohort (G and H, n = 414; 73-88 y). Cognitively unimpaired comprised of cognitively healthy controls and participants with subjective cognitive decline. Cognitively impaired comprised of participants with mild cognitive impairment and Alzheimer disease dementia. AUC indicates area under the curve; and NFL, neurofilament light chain.

The results from the logistic regression models in BioFINDER of all tested single and combined biomarkers are shown in eTables 4, 5, and 6 in the Supplement (including AUC and AIC values). The receiver operating characteristic curves and AUCs of selected biomarkers for predicting Aβ positivity are shown in Figure 2A and B (sensitivity, specificity, and cutoffs are shown in Table 2). The plasma Aβ42/Aβ40 ratio predicted Aβ positivity with an AUC of 0.77 (95% CI, 0.74-0.81) in the whole BioFINDER population. Using plasma Aβ42 and Aβ40 as separate predictors in a logistic regression resulted in a slightly but significantly better AUC (0.80; 95% CI, 0.77-0.83; *P* = .01) and a better model fit (ΔAIC, –66). We also tested the accuracy of the biomarkers in different age groups and in those with and without cognitive impairment (Figure 3; eTable 4 and eTable 6 in the Supplement), with similar results (AUC ±0.02 compared with the total population).

**Table 2.** Sensitivity and Specificity for Aβ Status in the BioFINDER Cohort and the Validation Cohort

Plasma Biomarkers	Cutoff^a^	% (95% CI)	
		Sensitivity	Specificity
BioFINDER cohort			
Aβ42/Aβ40 ratio	0.065	75 (68-80)	72 (65-77)
Aβ42, Aβ40	0.45	73 (65-78)	76 (68-80)
Aβ42, Aβ40, tau	0.36	86 (78-90)	68 (61-72)
Aβ42, Aβ40, NFL	0.38	84 (76-88)	70 (62-74)
Aβ42, Aβ40, *APOE*	0.29	88 (82-92)	68 (58-72)
Aβ42, Aβ40, tau, NFL, *APOE*	0.52	73 (64-78)	86 (77-89)
Validation cohort			
Aβ42/Aβ40 ratio	0.065	70 (61-80)	73 (61-81)
Aβ42, Aβ40	0.45	89 (80-95)	69 (54-81)
Aβ42, Aβ40, tau	0.36	89 (74-94)	64 (49-74)

Abbreviations: Aβ, β-amyloid; NFL, neurofilament light chain.

^a^ Cutoffs were determined based on the highest Youden index (sensitivity + specificity – 1) for Aβ positivity in the BioFINDER cohort. The cutoffs were then replicated in the validation cohort. Cutoffs are from the probabilities from the corresponding logistic regression models, except for Aβ42/Aβ40 where the actual ratio of the biomarker levels constitute the cutoff. Aβ status (reference standard) was determined using the cerebrospinal fluid Aβ42/40 ratio (<0.059). The 95% CIs were computed using 2000 stratified bootstrap replicates. Neurofilament light chain and *APOE* genotype were not available in the validation cohort.

### Aβ Detection With Additional Predictors

The accuracy of predicting Aβ status was further examined by adding *APOE* genotype, and plasma levels of tau, NFL, and NFH to plasma Aβ42 and Aβ40 in logistic regression models (Figure 2A-B). When adding plasma tau, AUC increased nonsignificantly to 0.81 (95% CI, 0.78-0.84) and further improved the model fit (ΔAIC, –27). However, when instead adding *APOE* genotype to plasma Aβ42 and Aβ40, AUC increased significantly from 0.80 to 0.85 (95% CI, 0.82-0.88; *P* < .001; Figure 2A-B; eTable 4 in the Supplement). Adding plasma tau to plasma Aβ42, Aβ40, and *APOE* increased the AUC slightly to 0.86 (95% CI, 0.83-0.88; ΔAIC, –20). A further slight increase was seen when adding plasma NFL to plasma Aβ42, Aβ40, tau, and *APOE* (AUC, 0.87; 95% CI, 0.84-0.89; ΔAIC –16; Figure 2A-B). The results were similar in CU and cognitively impaired participants, respectively, except that plasma tau and NFL were not a significant predictor in the cognitively impaired group (eTable 4 in the Supplement). The results were also similar when the CSF P-tau/Aβ42 ratio was used to define Aβ positivity (eFigure 3 in the Supplement). Plasma NFH did not contribute to Aβ prediction in addition to plasma Aβ42 and Aβ40 (eTable 5 in the Supplement).

### Independent Validation Cohort

Among the 237 study participants in the independent validation cohort, mean (SD) age was 66 (10) years with a range of 23 to 85 years, and 120 (50.6%) were female. The demographic characteristics are shown in eTable 7 in the Supplement and the accuracy of the plasma assays in Figure 2C and D. The AUC for plasma Aβ42 and Aβ40 to predict Aβ positivity was 0.86 (95% CI, 0.81-0.91) when applying the estimates from the model established in BioFINDER (compared with an AUC of 0.80, 95% CI 0.77-0.83 in BioFINDER). When applying the BioFINDER model that included plasma Aβ42, Aβ40, and tau in the validation cohort, the AUC was slightly lower than when using only plasma Aβ42 and Aβ40 (AUC, 0.84; 95% CI, 79-89). With the alternative reference standard for Aβ status (CSF P-tau/Aβ42 ≥ 0.022; eFigure 4 in the Supplement), the accuracy was slightly lower for plasma Aβ42 and Aβ40 (AUC, 0.83; 95% CI, 0.78-0.89) but still better than the corresponding results in the BioFINDER cohort (AUC, 0.79; 95% CI, 0.76-0.82; eFigure 3 in the Supplement). Sensitivities and specificities using the cutoffs established in BioFINDER are shown in Table 2. Plasma NFH, NFL, and *APOE* genotype were not available in the validation cohort.

### Cost-Benefit Analysis

Finally, we performed a cost-benefit analysis (eFigure 5 in the Supplement) where we show a scenario in which 1000 Aβ-positive participants are included in a trial where the screening cost for Aβ PET is $4000 per participant.^43^ For example, using the highest Youden index cutoff (Table 2) for plasma Aβ42, Aβ40, and *APOE* reduces the number of PET scans by approximately 800 and lowers the PET costs by approximately $3.2 million (from a total cost of approximately $9.2 million).

## Discussion

In this study of 842 participants, we found that plasma Aβ42 and Aβ40 using the fully automated Elecsys platform detected abnormal levels of Aβ in the brain with an AUC of 0.80 (Figure 2A and B). The addition of *APOE* genotype increased the AUC significantly to 0.85 (Figure 2A and B). Plasma tau and NFL had a slight effect on the accuracy (AUC, +0.01 to 0.02; Figure 2A and B). The results were similar in cognitively impaired and unimpaired and older and younger participants (Figure 3), with the exception that plasma tau and NFL generally did not improve accuracy in addition to plasma Aβ and *APOE* genotype in cognitively impaired participants (eTable 4 in the Supplement). When applying the plasma Aβ42 and Aβ40 model from BioFINDER to the independent validation cohort (n = 237), the AUC was greater compared with BioFINDER (AUC, 0.86; 95% CI, 0.81-0.91), but no improvement was seen when adding plasma tau (Figure 2C and D).

Although previous studies have found associations between CSF and PET Aβ and plasma Aβ using different immunoassays,^7,12,13,14,44,45^ the present Elecsys assays produced among the best accuracies and they are the first fully automated assays to have these greater accuracies. In mass spectrometry–based techniques, 2 recent studies have provided overall better accuracies for plasma Aβ42/Aβ40 (AUC, 0.84-0.97 depending on population and reference standard).^19,20^ However, these are labor-intensive, time-consuming, low-throughput methods that currently are not feasible to implement in clinical practice on a large scale. Fully automated Elecsys assays, on the other hand, are already implemented in many clinical chemistry laboratories worldwide that provide analyses (eg, for primary care).

Historically, the ratio of plasma Aβ42 to Aβ40 has been used to optimize the concordance with CSF or PET Aβ. Here, Aβ40 acts as a reference peptide that accounts for interindividual variability in the overall Aβ production and CSF turnover. We found that instead of using the fixed ratio of Aβ42/Aβ40, both the model fit (AIC) and accuracy (AUC) were slightly but significantly improved when the model was adjusted for Aβ40 concentrations independent of Aβ42 (ie, used as a separate predictor in the logistic regression models) (AUC 0.77 vs 0.80; *P* = .01; ΔAIC –66; eTable 4 in the Supplement). As a single additional biomarker to Aβ42 and Aβ40, *APOE* genotype increased the accuracy most markedly, from AUC 0.80 to 0.85 (*P* < .001) (Figure 2A and B; eTable 4 in the Supplement). Plasma tau increased the AUC slightly, and provided a better model fit (ΔAIC –27), but clinically this is not comparable with the contribution CSF tau has combined with CSF Aβ42,^24^ and improved plasma tau assays are probably needed in the future, such as measurement of specifically phosphorylated tau,^46^ to increase the added value of plasma tau to plasma Aβ42 and Aβ40.

Despite the present and previous results showing relatively high correlations between plasma and CSF NFL (eTable 3 in the Supplement and the study by Hansson et al^47^), we saw a modest increase in accuracy in addition to plasma Aβ42 and Aβ40 (Figure 2; eTable 4 in the Supplement). Because NFL generally is late biomarker in the disease process and a non–AD specific biomarker for axonal degeneration,^15^ the poor result could be because most of the participants (513 of 842) were cognitively unimpaired and only 64 had AD dementia. Compared with plasma tau and NFL, plasma NFH had a poorer performance and did not improve accuracy (eTable 5 in the Supplement). However, 101 plasma NFH measurements were below the detection limit of the assays, and development of more sensitive plasma NFH assays is thus warranted to establish whether this biomarker could further improve the diagnostic performance of plasma Aβ.

Overall, the accuracies of the Aβ42 and Aβ40 assays are not sufficient to be used on their own as a clinical test of Aβ positivity; additional assay development is needed before this can be recommended, possibly together with other blood biomarkers and screening tools in diagnostic algorithms. In the present study, we showed that the Aβ assays perform similarly in CU populations with lower prevalence of Aβ positivity (Figure 3A and B; Aβ-positive prevalence, 29%). Nonetheless, further studies would be valuable in populations with lower prevalence of Aβ positivity, such as primary care settings, as well as more heterogeneous dementia cohorts with different neurodegenerative disorders. To some extent, the generalizability of the BioFINDER results has already been shown in the present study where the plasma Aβ42 and Aβ40 model established in BioFINDER could be applied in the independent validation cohort with better accuracy (AUC, 0.86 vs 0.80; Figure 2). This robust result is similar to what has been shown when using the Elecsys assays for CSF to establish a cutoff in one cohort and replicating it in a second cohort.^24^

### Limitations

Limitations of the present validation analysis include the lack of *APOE* data, the lack of improvement when replicating the model that included plasma tau, and the smaller population size, resulting in a lack of analyses in subpopulations. The latter was, however, tested in BioFINDER and the accuracies were similar in different subsamples including CU (Figure 3A and B) and younger participants (Figure 3E and F) where Aβ positivity might be more difficult to identify using alternative methods such as cognitive testing and age stratification.^40^

## Conclusions

From a practical perspective, we believe that the most advantageous future use of optimized blood Aβ assays is as a screening tool for identifying subjects at a higher risk of being Aβ positive. They could, for example, be applied as an initial test together with other noninvasive, cost-efficient tools that aid the decision about whom a general practitioner should refer for further investigation at memory clinics where CSF or PET and more extensive clinical assessment could be used to support the AD diagnosis. Another useful setting for the blood biomarkers are clinical AD trials enrolling Aβ-positive participants, where they can be used for prescreening to minimize the number of unnecessary (Aβ-negative) lumbar punctures and Aβ PET scans, as well as lowering the costs for the examinations up to 30% to 50% depending on the cutoff (eFigure 5 in the Supplement).^48^ Although further validation studies are needed, this illustrates the potential usefulness blood assays might have, especially considering the ongoing great need to recruit large cohorts for AD drug trials in preclinical and prodromal stages.

## Notes

Supplement. **eMethods.** **eResults.** **eTable 1.** Performance Characteristics of the Plasma Aβ42, Aβ40 and Tau Elecsys Assays **eTable 2.** Performance Characteristics of CSF and Plasma NFH Assays **eTable 3.** Associations Between Plasma and CSF Biomarkers. **eTable 4.** Area Under the Curves From Logistic Regression Models for Prediction of Aβ Positivity **eTable 5.** Plasma NfH as Additional Predictor for Aβ Positivity. **eTable 6.** Area Under the Curves From Logistic Regression Models for Prediction of Aβ Positivity in the Younger and Older Half of the BioFINDER Cohort. **eTable 7.** Demographic and Clinical Data of the German Validation Cohort **eFigure 1.** Correlations Between Plasma and CSF Biomarkers. **eFigure 2.** Plasma Biomarkers in Diagnostic Groups. **eFigure 3.** ROC Analysis of Plasma Biomarkers Using the Ratio of CSF P-tau/Aβ42 as Reference Standard in BioFINDER. **eFigure 4.** ROC Analysis of Plasma Biomarkers Using the Ratio of CSF P-tau/Aβ42 as Reference Standard in the Independent Validation Cohort. **eFigure 5.** Implementation of Plasma Aβ42, Aβ40 and APOE Genotype in an AD Trial Screening Scenario. **eReferences.**
