# Supplementary material for: Performance of Fully Automated Plasma Assays as Screening Tests for Alzheimer Disease–Related β-Amyloid Status
Source: JAMA Neurol. 2019 Jun 24;76(9):1060–9. doi: 10.1001/jamaneurol.2019.1632 (PMC6593637; doi:10.1001/jamaneurol.2019.1632)
Supplement: Supplement. — eMethods. eResults. eTable 1. Performance Characteristics of the Plasma Aβ42, Aβ40 and Tau Elecsys Assays eTable 2. Performance Characteristics of CSF and Plasma NFH Assays eTable 3. Associations Between Plasma and CSF Biomarkers. eTable 4. Area Under the Curves From Logistic Regression Models for Prediction of Aβ Positivity eTable 5. Plasma NfH as Additional Predictor for Aβ Positivity. eTable 6. Area Under the Curves From Logistic Regression Models for Prediction of Aβ Positivity in the Younger and Older Half of the BioFINDER Cohort. eTable 7. Demographic and Clinical Data of the German Validation Cohort eFigure 1. Correlations Between Plasma and CSF Biomarkers. eFigure 2. Plasma Biomarkers in Diagnostic Groups. eFigure 3. ROC Analysis of Plasma Biomarkers Using the Ratio of CSF P-tau/Aβ42 as Reference Standard in BioFINDER. eFigure 4. ROC Analysis of Plasma Biomarkers Using the Ratio of CSF P-tau/Aβ42 as Reference Standard in the Independent Validation Cohort. eFigure 5. Implementation of Plasma Aβ42, Aβ40 and APOE Genotype in an AD Trial Screening Scenario. eReferences. [file jamaneurol-76-1060-s001.pdf]

Supplementary Online Content

Palmqvist S, Janelidze S, Stomrud E, et al. Performance of fully automated plasma assays as screening tests for Alzheimer disease–related β-amyloid status. *JAMA Neurol*. Published online June 24, 2019. doi:10.1001/jamaneurol.2019.1632

eMethods.

eResults.

eTable 1. Performance Characteristics of the Plasma Aβ42, Aβ40 and Tau Elecsys Assays

eTable 2. Performance Characteristics of CSF and Plasma NfH AssayseTable 1

eTable 3. Associations Between Plasma and CSF Biomarkers.

eTable 4. Area Under the Curves From Logistic Regression Models for Prediction of Aβ Positivity

eTable 5. Plasma NfH as Additional Predictor for Aβ Positivity.

eTable 6. Area Under the Curves From Logistic Regression Models for Prediction of Aβ Positivity in the Younger and Older Half of the BioFINDER Cohort.

eTable 7. Demographic and Clinical Data of the German Validation Cohort

eFigure 1. Correlations Between Plasma and CSF Biomarkers.

eFigure 2. Plasma Biomarkers in Diagnostic Groups.

eFigure 3. ROC Analysis of Plasma Biomarkers Using the Ratio of CSF P-tau/Aβ42 as Reference Standard in BioFINDER.

eFigure 4. ROC Analysis of Plasma Biomarkers Using the Ratio of CSF P-tau/Aβ42 as Reference Standard in the Independent Validation Cohort.

eFigure 5. Implementation of Plasma Aβ42, Aβ40 and APOE Genotype in an AD Trial Screening Scenario.

eReferences.

This supplementary material has been provided by the authors to give readers additional information about their work.

©Palmqvist S et al. *JAMA Neurology*.

eMethods

Participants of the BioFINDER cohort

The healthy elderly participants without cognitive symptoms (n=318) were originally enrolled from the population-based EPIC cohort.[1] The inclusion criteria were (1) age ≥60 years old, (2) Mini-Mental State Examination (MMSE) score of 28-30 points, and (3) fluent in Swedish. The exclusion criteria were (1) presence of subjective cognitive impairment, (2) significant neurologic disease (for example, stroke, Parkinson's disease, multiple sclerosis), (3) severe psychiatric disease (for example, severe depression or psychotic syndromes), and (4) dementia or mild cognitive impairment (MCI).

The inclusion criteria for patients with subjective cognitive decline (SCD; n=195) or MCI (n=265) were that they (1) were referred to participating memory clinics because of cognitive complaints; (2) did not fulfill the criteria for dementia; (3) had a MMSE score of 24 to 30 points; (4) were aged 60 to 80 years; and (5) were fluent in Swedish. The exclusion criteria were (1) cognitive impairment that without doubt could be explained by another condition (other than prodromal dementia), such as brain tumor, brain trauma etc.; (2) severe somatic disease; and (3) refusing lumbar puncture or neuropsychological testing.

The patients with Alzheimer's disease (AD) dementia (n=64) fulfilled the NIA-AA criteria for probable AD[2] and in the present study we also required that all were Aβ positive.

In agreement with the latest research criteria for AD[3], the healthy elderly participants and patients with SCD were classified as cognitively unimpaired (CU, n=513).

Participants of the independent validation cohort

All participants of this study were enrolled between 2000 and 2006 at two clinical sites in Germany (at the Geriatric and Rehabilitation Clinic of the Henriettenstift in Hannover and at the Neurological Clinic of the University of Ulm), as part of a prospective validation study of new biomarkers in CSF, blood and urine for the early diagnosis of AD. The inclusion criteria for the mild AD dementia group were: (1) fulfilling the DSM-IV[4] and NINCDS-ADRDA criteria for probable AD[5], (2) MMSE score ≥14 and (3) Hachinski Ischaemia Scale score less than 4. The participants of the MCI group met the MCI criteria by Petersen[6] and fulfilled the following inclusion criteria: (1) memory complaints and difficulties that were verified by an informant who knew the patient well, (2) isolated episodic memory loss, (3) memory impairment of insidious onset and not caused by endogenous factors, (4) additional cognitive impairment not sufficient to warrant a diagnosis of dementia according to DSM-IV[4], (5) not significantly impaired activities of daily living, (6) global CDR score of 0.5[7], and (7) Hachinski Ischaemia Scale score less than 4. The inclusion criteria for the cognitively unimpaired (CU) controls were (1) no psychiatric diagnosis (DSM-IV axis 1 diagnosis), (2) no evidence of memory or other cognitive impairment, verified by a reliable informant and/or psychometric testing (3) no previous or current history of inflammatory, neoplastic or traumatic disorder of the peripheral or central nervous system, (4) no previous or current history of degenerative or ischaemic disorders of the nervous central system, and (5) no previous or current history of systemic disorders that may impact CSF analysis.

For all diagnostic groups, the following exclusion criteria were used: (1) DSM-IV axis 1 diagnosis other than those specified in the inclusion criteria, (2) anticoagulant drugs or continuously (>3 months) treatment with COX-2 inhibitors, (3) treatment with antidepressants, antipsychotics or benzodiazepines for 30 days prior to sample collection, and (4) treatment with any drugs that may interfere with cognitive testing.

©Palmqvist S et al. *JAMA Neurology*.

CSF and plasma procedures and analysis in BioFINDER

CSF and plasma Aβ(1–42), Aβ(1–40) and total tau

The cerebrospinal fluid (CSF) and plasma samples were analyzed using the Elecsys Aβ1–42 (Aβ42), Aβ1–40(Aβ40) and total tau (tau) immunoassays on a **cobas e** 601 analyzer (software version 05.02) at the Clinical Neurochemistry Laboratory, University of Gothenburg, Sweden. CSF and plasma Aβ42 and tau assays were performed as previously described[8,9] with some modifications implemented for plasma samples. The Elecsys method is an antibody-based technique (which gives high analytical sensitivity), a sandwich immunoassay, based on one capture and one detection antibody (which increases the specificity). For that reason, it is performed on neat plasma, without any cleanup or pre-treatment step. In comparison to the CSF assays, other calibrators and controls were used for the plasma assay to overcome the different sample matrix and the different analyte levels in plasma. Chemically synthesized tau-antigen (same as for the CSF assay) was spiked into a protein-containing TRIS-buffer in concentrations of approx. 0, 30, 100, 500 and 5000 pg/ml. Chemically synthesized Aβ42-antigen (same as for the CSF assay) was spiked into horse serum in concentrations of approx. 0, 20, 50, 250 and 1200 pg/ml.

For Aβ40 analysis in plasma, 50 µL of sample, a biotinylated monoclonal Aβ40 specific antibody (23C2) and a monoclonal β-Amyloid-specific antibody (3D6) labeled with a ruthenium complex were first co-incubated for 9 minutes to form a sandwich complex comprising the biotinylated antibody, Aβ40 and the ruthenylated antibody. In the second incubation step (9 minutes), streptavidin-coated microparticles (Elecsys® beads) were added to the mixture of the first incubation step and, as a result, the complex comprising the biotinylated antibody, Aβ40 and the ruthenylated antibody became bound to the solid phase via interaction of biotin and streptavidin. The reaction mixture was aspirated into the measuring cell where the microparticles were magnetically captured onto the surface of the electrode. Unbound substances were then removed with ProCell/ProCell M. Application of a voltage to the electrode then induced chemiluminescent emission which was measured by a photomultiplier. Samples concentrations were determined from a 5-point calibration curve. To generate calibrators, phosphate-buffered saline containing 0.4 g/L BSA was spiked with a chemically synthesized Aβ40 antigen, which contained both antibody recognizing epitopes 1-12 and 25-40. Following levels of Aβ40 were produced: 0, 250, 500, 2500 and 10.000 pg/ml. In comparison with the plasma application for the Aβ40 determination in CSF the same reagents were used, but only 20 µL of sample were pipetted to the reaction mixture with the labeled antibodies and the target values of the calibrators were changed to 0, 1, 3, 9, and 30 ng/ml.

All calibrator sets were frozen at -80°C before use. Aβ42, Aβ40 and tau were measured individually from the same aliquot. The limits of quantification for Aβ42, Aβ40 and tau were 5.8 pg/mL, 9 pg/mL and 1 pg/mL, respectively. Intra-assay and inter-assay coefficient of variation are shown in eTable 1.

CSF and plasma NfL and NfH

CSF and plasma neurofilament light chain (NfL) were analyzed at the Clinical Neurochemistry Laboratory, University of Gothenburg, Sweden as previously described.[10] CSF and plasma neurofilament heavy chain (NfH) were analysed using ELISA kit at Euroimmun AG, Lübeck, Germany according to manufacturer's recommendations. The lower limit of detection for CSF and plasma NfH were 27 pg/ml and 1.7 pg/ml, respectively with intra-assay and inter-assay coefficient shown in eTable 2.

CSF and blood procedures in the validation cohort

Blood samples were collected at the same time as CSF samples. Lumbar puncture (LP) was performed according to the standards implemented in the clinical centers. CSF was collected into a neutral polypropylene tube (Sarstedt, 60.541.545) or similar device and was cooled on ice. Immediately, at the latest 30 minutes after puncture, cells were removed by centrifugation of CSF for 10 minutes at 2000 g and 4°C. The supernatant was transferred into a further polypropylene tube for freezing at -80°C. After transfer to Roche the tubes were thawed and aliquoted in polypropylene tubes (Sarstedt, 72.730.003) for long time storage at -80°C. All CSF samples had gone through two freeze-thaw cycles before the analysis.

Blood samples were also collected according to a standardized protocol. For each study participant, blood was collected in an EDTA-plasma tube (S-Monovette® Sarstedt) and centrifuged (2000g, +4°C) for 10 min. Following centrifugation, the plasma was immediately frozen at -80°C in Sarstedt Monovette tubes. After transfer to Roche the tubes were thawed and aliquoted in polypropylene tubes (Sarstedt, 72.730.003) for long time storage at -80°C. All plasma samples have gone through two freeze-thaw cycle before the analysis.

The current standardized protocol is consistent with recent findings that blood need to be centrifuged within 1 h and frozen shortly thereafter, however, up to three freeze/thaw cycles and five tube transfers do not affect plasma Aβ and tau values.[11] Plasma and CSF Aβ42, Aβ40, total tau (tau) and phosphorylated tau (P-tau; only in CSF) were analyzed using the Elecsys immunoassays on a **cobas e** 601 analyzer at Roche Diagnostics, Penzberg, Germany.

CSF and plasma Aβ42, Aβ40 and tau were analyzed the same way as in the BioFINDER cohort (see description under "CSF and plasma procedures and analysis in BioFINDER" in this online supplement).

Statistical analysis

For 101 samples, plasma NfH levels were below the detection limit of the assay. These samples were assigned NfH values equal to the lower detection limit of the assay. Correlations between plasma and CSF biomarkers were examined with Spearman's correlation test. Group differences in the biomarker levels were first tested with one-way ANOVA and, when statistically significant, further investigated in univariate general linear models (GLM) adjusting for potential confounders with age and sex included as covariates. For group comparisons, p-values were corrected using the Bonferroni method. 95% confidence intervals (CI) of the AUCs were determined according to the DeLong method.[12]

©Palmqvist S et al. *JAMA Neurology*.

eResults

APOE genotype analysis

Four different APOE variables were created: Presence of A) ε2/ε2 or ε2/ε3, B) ε3/ε3, C) ε2/ε4 or ε3/ε4, and D) ε4/ε4. *APOE* ε3/ε3 was the reference category in the logistic regression analysis. This grouping produced an AUC of 0.76 (95% CI 0.73-0.79; AIC 945) when predicting Aβ positivity in the entire population (n=842). This grouping of *APOE* genotype was slightly better than alternative variants, such as a dichotomous coding of ε4 presence (0 or 1; AUC 0.74, 95% CI 0.71-0.77; AIC 962) and number of ε4 alleles (0, 1 or 2; AUC 0.75, 95% CI 0.72-0.78; AIC 947).

©Palmqvist S et al. *JAMA Neurology*.

eReferences

1. Haftenberger M, Schuit AJ, Tormo MJ, et al. Physical activity of subjects aged 50-64 years involved in the European Prospective Investigation into Cancer and Nutrition (EPIC). *Public Health Nutr.* 2002;5(6B):1163-1176.
2. McKhann GM, Knopman DS, Chertkow H, et al. The diagnosis of dementia due to Alzheimer's disease: Recommendations from the National Institute on Aging and the Alzheimer's Association workgroup. *Alzheimers Dement.* 2011.
3. Jack CR, Jr., Bennett DA, Blennow K, et al. NIA-AA Research Framework: Toward a biological definition of Alzheimer's disease. *Alzheimers Dement.* 2018;14(4):535-562.
4. American Psychiatric Association A. *Diagnostic and Statistical Manual of Mental Disorders, 4th ed.* Washington, DC, USA: American Psychiatric Association (APA); 1994.
5. McKhann G, Drachman D, Folstein M, Katzman R, Price D, Stadlan EM. Clinical diagnosis of Alzheimer's disease: report of the NINCDS-ADRDA Work Group under the auspices of Department of Health and Human Services Task Force on Alzheimer's Disease. *Neurology.* 1984;34(7):939-944.
6. Petersen RC, Smith GE, Waring SC, Ivnik RJ, Tangalos EG, Kokmen E. Mild cognitive impairment: clinical characterization and outcome. *Arch Neurol.* 1999;56(3):303-308.
7. Morris JC. The Clinical Dementia Rating (CDR): current version and scoring rules. *Neurology.* 1993;43(11):2412-2414.
8. Bittner T, Zetterberg H, Teunissen CE, et al. Technical performance of a novel, fully automated electrochemiluminescence immunoassay for the quantitation of beta-amyloid (1-42) in human cerebrospinal fluid. *Alzheimers Dement.* 2016;12(5):517-526.
9. Hansson O, Seibyl J, Stomrud E, et al. CSF biomarkers of Alzheimer's disease concord with amyloid-beta PET and predict clinical progression: A study of fully automated immunoassays in BioFINDER and ADNI cohorts. *Alzheimers Dement.* 2018.
10. Hansson O, Janelidze S, Hall S, et al. Blood-based NfL: A biomarker for differential diagnosis of parkinsonian disorder. *Neurology.* 2017;88(10):930-937.
11. Rozga M, Bittner T, Batrla-Utermann R, Karl J. Impact of pre-analytical sample handling on Elecsys® Aβ40, Aβ42 and tTau immunoassays in plasma. 11th Clinical Trials on Alzheimer’s disease (CTAD) 2018; Barcelona, Spain.
12. DeLong ER, DeLong DM, Clarke-Pearson DL. Comparing the areas under two or more correlated receiver operating characteristic curves: a nonparametric approach. *Biometrics.* 1988;44(3):837-845.
13. Blennow K, Mattsson N, Scholl M, Hansson O, Zetterberg H. Amyloid biomarkers in Alzheimer's disease. *Trends Pharmacol Sci.* 2015;36(5):297-309.
14. Insel PS, Palmqvist S, Mackin RS, et al. Assessing risk for preclinical beta-amyloid pathology with APOE, cognitive, and demographic information. *Alzheimers Dement (Amst).* 2016;4:76-84.

©Palmqvist S et al. *JAMA Neurology.*

eFigures

eFigure 1

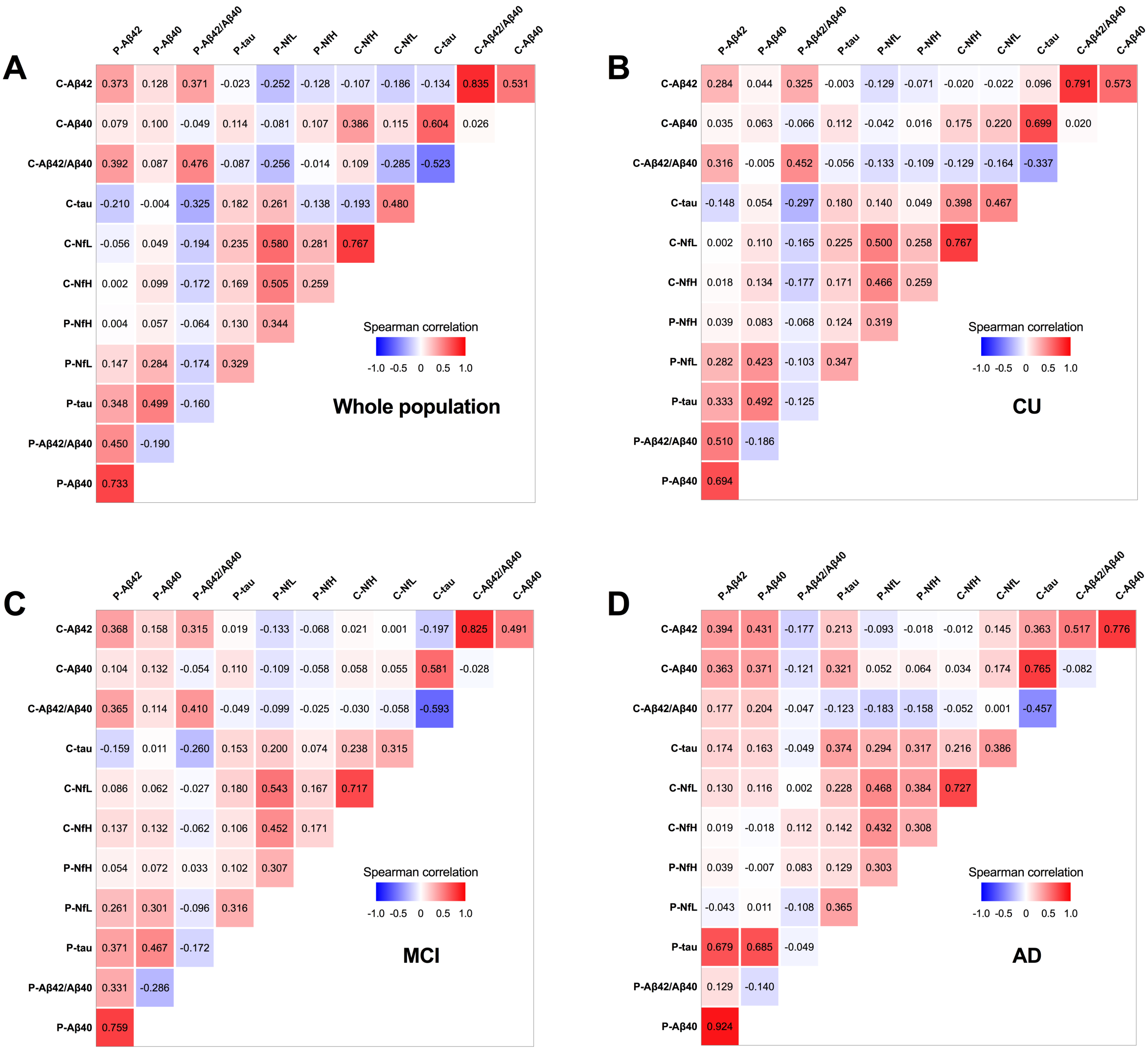

Correlations between plasma and CSF biomarkers. Heatmaps showing Spearman correlation coefficients between plasma (P) and CSF (C) biomarkers in the whole population (**A**) and in the CU (**B**), MCI (**C**) and AD (**D**) groups.

©Palmqvist S et al. *JAMA Neurology*.

eFigure 2

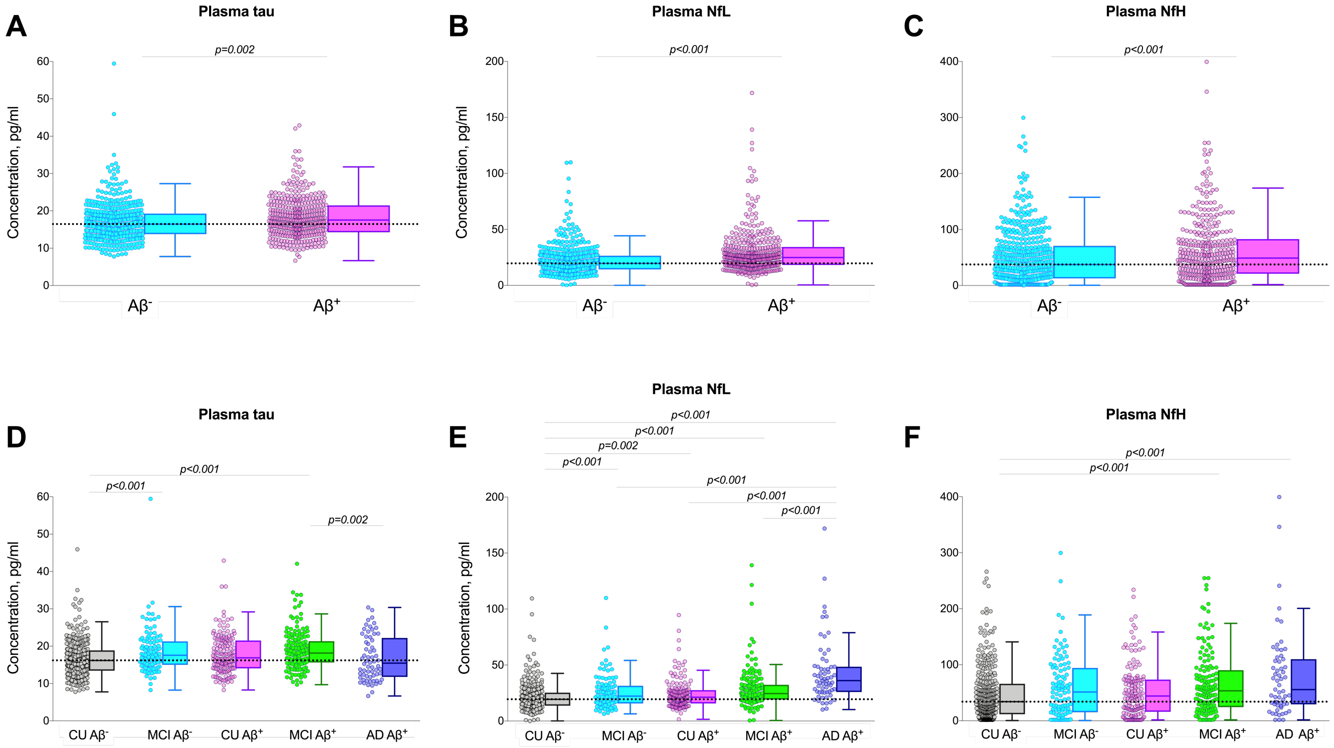

Plasma biomarkers in diagnostic groups. Plasma levels of tau (**A**), NfL (**B**) and NfH (**C**) in the Aβ+ (CSF Aβ42/Aβ40 ≤0.059) and Aβ- (CSF Aβ42/Aβ40>0.059) groups. Plasma levels of tau (**D**), NfL (**E**) and NfH (**F**) in cognitively unimpaired controls and MCI patients with normal (CU Aβ-, MCI Aβ-) and abnormal Aβ status (CU Aβ+, MCI Aβ+) and patients with AD dementia (AD Aβ+). The dotted lines indicate median levels in the CU Aβ- group. One case with NfL concentration of 728 pg/ml (CU Aβ+ group) and 3 cases with NfH concentrations of 700, 573 and 456 pg/ml (CU Aβ- group) are not shown. P values are from Student t test (**A-C**) or one-way ANOVA (**D-F**); statistical significance was set to p<0.005 (0.05/10) to account for Bonferroni correction. Nfl and NfH values were ln-transformed before the analysis. The significant findings were very similar when adjusting for age and sex except that there were no differences in NfL levels between AD Aβ+ and MCI Aβ- (p=0.033) and between CU Aβ- and CU Aβ+ (p=0.008).

Abbreviations: AD, Alzheimer's disease; CSF, cerebrospinal fluid. CU, cognitively unimpaired; MCI, mild cognitive impairment; NfH, neurofilament heavy; NfL, neurofilament light.

©Palmqvist S et al. *JAMA Neurology*.

eFigure 3

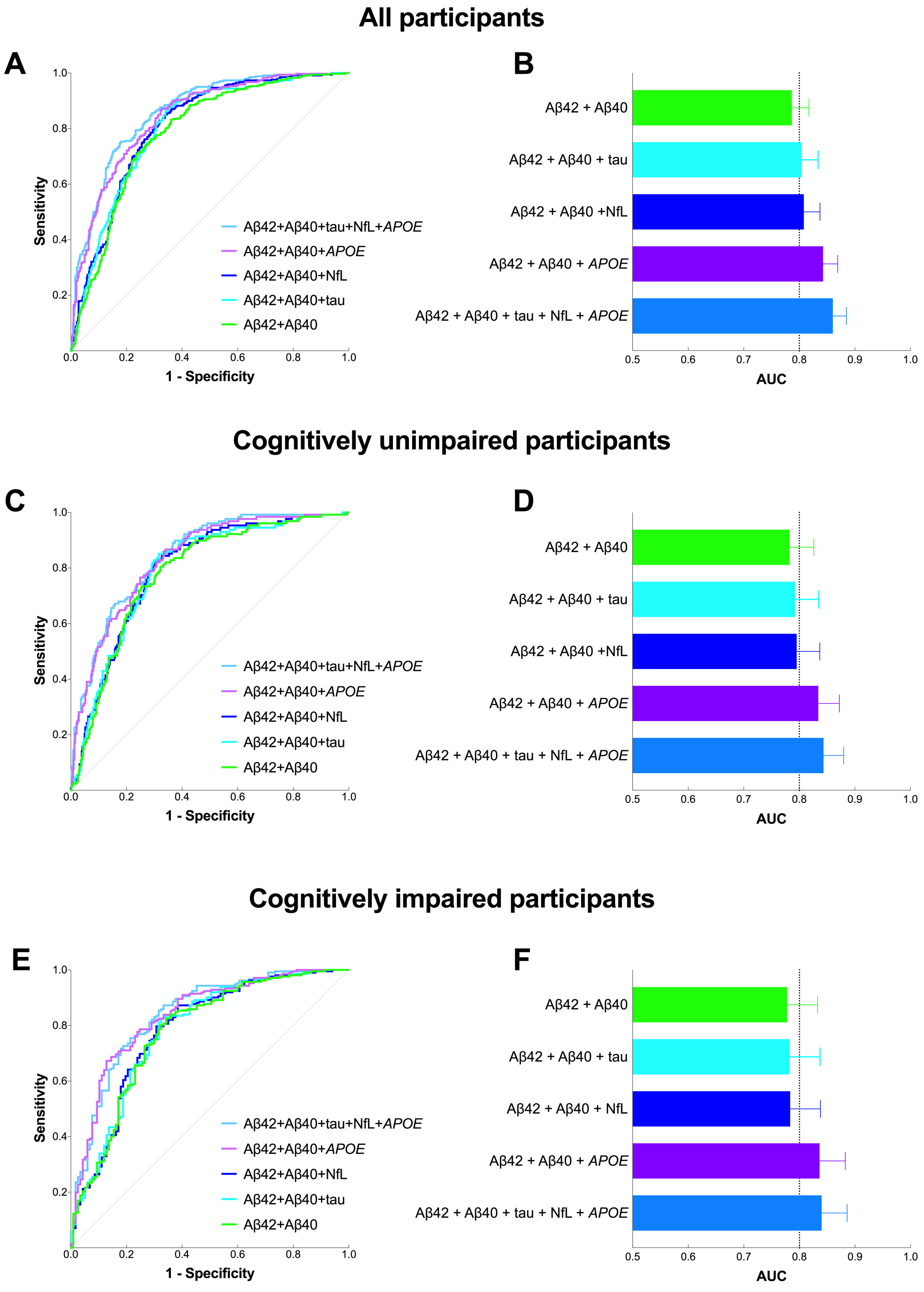

ROC analysis of plasma biomarkers using the ratio of CSF P-tau/Aβ42 as reference standard in BioFINDER. ROC curves and corresponding AUCs for plasma Aβ together with the additional predictors, *APOE*, plasma tau and NfL, to assess accuracy when predicting AD biomarker positivity (CSF P- tau/Aβ42 ≥0.022) in the whole population (A and B, n=842), CU (C and D, n=513) and cognitively impaired (E and F, n=329). Error bars are shown as 95% CI.
Abbreviations: AUC, area under the curve; CI, confidence interval; NfL, neurofilament light; P-tau, phosphorylated tau; ROC, receiver operating characteristic.

©Palmqvist S et al. *JAMA Neurology*.

eFigure 4

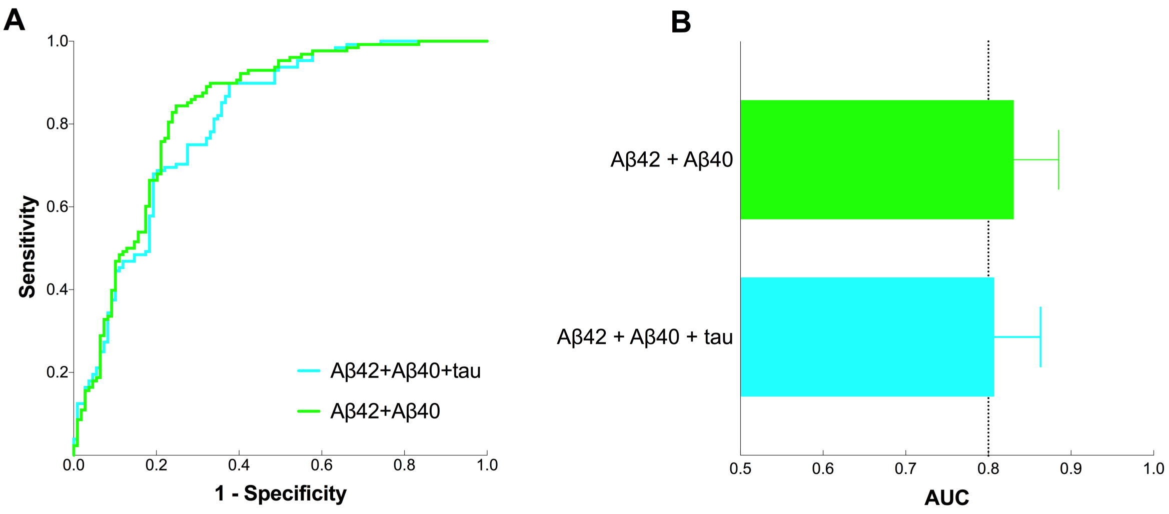

ROC analysis of plasma biomarkers using the ratio of CSF P-tau/Aβ42 as reference standard in the independent validation cohort. ROC curves and corresponding AUCs for plasma Aβ together with plasma tau to assess accuracy when predicting AD biomarker positivity (CSF P- tau/Aβ42 ≥0.022) in the whole validation population (n=237). Error bars are shown as 95% CI.

Abbreviations: AUC, area under the curve; CI, confidence interval; P-tau, phosphorylated tau; ROC, receiver operating characteristic

©Palmqvist S et al. *JAMA Neurology*.

eFigure 5

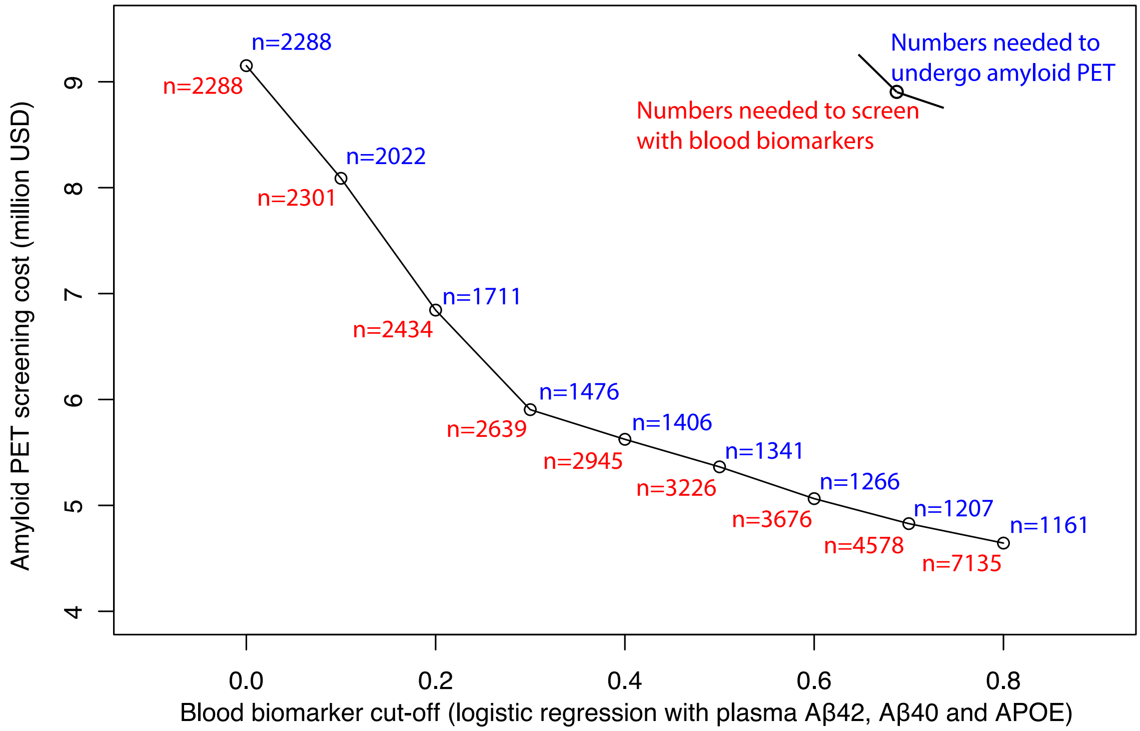

Implementation of plasma Aβ42, Aβ40 and *APOE* genotype in an AD trial screening scenario. Here, we assumed the same prevalence of Aβ positivity as in the BioFINDER study (44%) and a trial design that required 1000 Aβ+ subjects to be enrolled using Aβ PET to verify the Aβ status (with an approximate cost of 4000 USD per PET scan[13,14]). The y-axis shows the total Aβ PET cost and the x-axis the biomarker cut-off (probability of being Aβ positive according to logistic regression model using plasma Aβ42, Aβ40 and *APOE* genotype). The line in the graph shows the PET screening cost as a function of the plasma biomarker cut-offs, with number needed to screen with a blood test in red on the left side (the pre-screening process) and number needed to undergo an Aβ PET scan to verify the Aβ status in blue on the right side (normal trial screening process). The cost for the plasma analysis is not yet known and could therefore not be included.

©Palmqvist S et al. *JAMA Neurology*.

eTABLES

eTable 1. Performance characteristics of the plasma Aβ42, Aβ40 and Tau Elecsys assays

	Concentration (pg/ml)	Intra-assay CV (%)	Inter-assay CV (%)
**Aβ42**			
Sample 1	22	1.6	3.0
Sample 2	735	0.9	1.3
Sample 3	2986	1.0	1.3
**Aβ40**			
Sample 1	470	1.1	1.1
Sample 2	1330	1.4	1.1
Sample 3	6710	0.8	0.8
**Tau**			
Sample 1	10.3	1.3	2.1
Sample 2	826	0.9	1.0
Sample 3	3561	1.0	2.0

eTable 2. Performance characteristics of CSF and plasma NfH assays

	Intra-assay		Inter-assay	
	Concentration (ng/ml)	CV (%)	Concentration (ng/ml)	CV (%)
**CSF NfH**				
Sample 1	0.8	3.4	0.8	4.1
Sample 2	1.9	2.2	2.0	6.4
Sample 3	5.5	3.1	5.2	7.9
**Plasma NfH**				
Sample 1	71	4.0	71	4.7
Sample 2	351	6.0	312	6.9
Sample 3	816	4.7	615	10.6

©Palmqvist S et al. *JAMA Neurology*.

eTable 3. Associations between plasma and CSF biomarkers.

	Total<br>n=842	CU<br>n=513	MCI<br>n=265	AD<br>n=64
Aβ42	0.373***	0.284***	0.368***	0.395**
Aβ40	0.100**	0.063	0.132*	0.371**
T-Tau	0.182**	0.180***	0.153*	0.374**
Aβ42/Aβ40	0.476**	0.452***	0.410***	-0.047
NfL[a]	0.580***	0.500***	0.543***	0.468***
NfH[b]	0.259***	0.259***	0.171**	0.308*

Data are shown as rho (p) from Spearman correlation; * p<0.05, ** p<0.01, *** p<0.001.
Additional correlations can be found in eFigure 1. Abbreviations: NfH, neurofilament heavy chain; NfL, neurofilament light chain.
[a]Data were missing for CSF sample from 1 study participants. [b]Data were missing for from 21 study participants.
Abbreviations: AD, Alzheimer's disease; CU, cognitively unimpaired; MCI, mild cognitive impairment.

©Palmqvist S et al. *JAMA Neurology*.

eTable 4. Area under the curves from logistic regression models for prediction of Aβ positivity.

Plasma biomarkers	AUC (95% CI)<br>Whole population<br>(n=842)	AUC (95% CI)<br>Cognitively unimpaired<br>(n=513)	AUC (95% CI)<br>Cognitively impaired<br>(n=329)
Aβ42	0.71 (0.68-0.75)<br>AIC: 1040	0.71 (0.66-0.76)<br>AIC: 570	0.72 (0.66-0.78)<br>AIC: 374
Aβ40	0.54 (0.50-0.58)<br>AIC: 1148	0.52 (0.46-0.58)<br>AIC: 618<br>non-significant	0.57 (0.51-0.64)<br>AIC: 411
Tau	0.56 (0.52-0.60)<br>AIC: 1148	0.57 (0.51-0.63)<br>AIC: 611	0.50 (0.44-0.57)<br>AIC: 420<br>tau non-significant
NfL	0.64 (0.61-0.68)<br>AIC: 1123	0.58 (0.53-0.64)<br>AIC=609	0.62 (0.55-0.68)<br>AIC: 417<br>NfL non-significant
Aβ42/Aβ40 ratio	0.77 (0.74-0.81)<br>AIC: 1018	0.78 (0.73-0.82)<br>AIC: 545	0.75 (0.69-0.81)<br>AIC: 383
Aβ42, Aβ40	0.80 (0.77-0.83)<br>AIC: 952	0.78 (0.74-0.83)<br>AIC: 533	0.80 (0.74-0.85)<br>AIC: 341
Aβ42, tau	0.77 (0.74-0.81)<br>AIC: 965	0.77 (0.72-0.81)<br>AIC: 536	0.75 (0.69-0.81)<br>AIC: 362
Aβ42, NfL	0.78 (0.74-0.81)<br>AIC: 983	0.77 (0.72-0.81)<br>AIC: 543	0.75 (0.69-0.80)<br>AIC: 370
Aβ42, APOE	0.82 (0.79-0.84)<br>AIC: 887	0.81 (0.77-0.85)<br>AIC: 497	0.81 (0.76-0.86)<br>AIC:331
Aβ42, Aβ40, tau	0.81 (0.78-0.84)<br>AIC: 925	AUC 0.80 (0.76-0.84)<br>AIC: 521	0.80 (0.75-0.86)<br>AIC: 341<br>tau non-significant
Aβ42, Aβ40, NfL	0.82 (0.79-0.85)<br>AIC: 921	0.80 (0.77-0.85)<br>AIC: 521	0.81 (0.75-0.86)<br>AIC: 340<br>NfL non-significant
Aβ42, Aβ40, APOE	0.85 (0.82-0.88)<br>AIC: 831	0.84 (0.80-0.88)<br>AIC: 466	0.84 (0.79-0.89)<br>AIC: 315
Aβ42, Aβ40, tau, NfL	0.83 (0.80-0.85)<br>AIC: 907	0.81 (0.76-0.84)<br>AIC: 513	0.81 (0.75-0.86)<br>AIC: 341<br>tau and NfL non-significant
Aβ42, Aβ40, tau, APOE	0.86 (0.83-0.88)<br>AIC: 811	0.85 (0.81-0.88)<br>AIC: 457	0.84 (0.79-0.89)<br>AIC: 315<br>tau non-significant
Aβ42, Aβ40, NfL, APOE	0.86 (0.84-0.89)<br>AIC: 805	0.85 (0.81-0.88)<br>AIC: 457	0.84 (0.79-0.89)<br>AIC: 314<br>NfL non-significant
Aβ42, Aβ40, tau, NfL, APOE	0.87 (0.84-0.89)<br>AIC: 795	0.85 (0.82-0.89)<br>AIC: 451	0.84 (0.79-0.89)<br>AIC: 315<br>tau and NfL non-significant

AUCs (95% CIs) from logistic regression models for predicting Aβ positivity (CSF Aβ42/Aβ40 ≤0.059). Plasma NfH data are shown separately in eTable 5 due to missing data in 21 cases. AIC shows the model fit in relation to the number of variables (lower = better within the same population). Cognitively impaired consisted of MCI and AD participants (see Table 1). Abbreviations: AIC, Akaike information criterion; AUC, area under the ROC

©Palmqvist S et al. *JAMA Neurology*.

curve; CI, confidence interval; CU, cognitively unimpaired; MCI, mild cognitive impairment; NfH, neurofilament heavy chain; NfL, neurofilament light chain; ROC, receiver operating characteristic.

eTable 5. Plasma NfH as additional predictor for Aβ positivity.

Plasma biomarkers	AUC (95% CI) Whole population (n=821)	AUC (95% CI) Cognitively unimpaired (n=504)	AUC (95% CI) Cognitively impaired (n=258)
Aβ42	0.71 (0.68-0.75)<br>AIC: 1016	0.70 (0.65-0.75)<br>AIC: 562	0.72 (0.66-0.78)<br>AIC: 362
Aβ40	0.53 (0.49-0.58)<br>AIC: 1120	0.52 (0.46-0.57)<br>AIC: 607<br>non-significant	0.57 (0.50-0.63)<br>AIC: 400
tau	0.57 (0.53-0.61)<br>AIC: 1118	0.57 (0.51-0.62)<br>AIC: 601	0.49 (0.43-0.56)<br>AIC: 408<br>non-significant
NfH	0.57 (0.53-0.61)<br>AIC: 1122	0.54 (0.49-0.60)<br>AIC: 607<br>non-significant	0.55 (0.48-0.62)<br>AIC:406<br>non-significant
Aβ42, Aβ40, *APOE*	0.85 (0.82-0.87)<br>AIC: 815	0.84 (0.80-0.88)<br>AIC: 460	0.84 (0.79-0.88)<br>AIC: 307
Aβ42, Aβ40, *APOE*, NfH	0.85 (0.82-0.88)<br>AIC: 814<br>NfH non-significant	0.84 (0.80-0.88)<br>AIC: 462<br>NfH not significant	0.84 (0.79-0.89)<br>AIC: 306<br>NfH not significant
Aβ42, Aβ40, *APOE*, tau	0.86 (0.83-0.88)<br>AIC: 796	0.85 (0.81-0.88)<br>AIC: 453	0.84 (0.79-0.89)<br>AIC: 307<br>tau non-significant
Aβ42, Aβ40, *APOE*, tau, NfH	0.86 (0.83-0.88)<br>AIC: 796<br>NfH not significant	0.85 (0.81-0.88)<br>AIC: 455<br>NfH not significant	0.84 (0.79-0.89)<br>AIC: 306<br>tau and NfH not significant

AUCs from logistic regression models for predicting Aβ positivity (CSF Aβ42/Aβ40 ≤0.059). The analyses were performed using the subset where plasma NfH data were available (n=821). All AD subjects were per definition Aβ+ and could therefore not be examined separately. Results are shown as areas under the ROC curves (95% CI). The models are sorted based on AUC in the whole population.
Abbreviations: AIC, akaike information criterion; AUC, area under the curve; CI, confidence interval; CU, cognitively unimpaired; MCI, mild cognitive impairment.

©Palmqvist S et al. *JAMA Neurology*.

eTable 6. Area under the curves from logistic regression models for prediction of Aβ positivity in the younger and older half of the BioFINDER cohort.

Plasma biomarkers	AUC (95% CI) younger (n=428)	AUC (95% CI) older (n=414)
Aβ42, Aβ40	0.81 (0.77-0.85)<br>AIC: 465	0.79 (0.75-0.84)<br>AIC: 465
Aβ42, Aβ40, tau	0.82 (0.78-0.86)<br>AIC: 456	0.81 (0.77-0.85)<br>AIC: 456
Aβ42, Aβ40, NfL	0.83 (0.79-0.87)<br>AIC: 451	0.81 (0.77-0.85)<br>AIC: 451
Aβ42, Aβ40, *APOE*	0.85 (0.82-0.89)<br>AIC: 413	0.86 (0.82-0.89)<br>AIC: 413
Aβ42, Aβ40, *APOE*, tau, NfL	0.87 (0.83-0.90)<br>AIC: 402<br>tau not significant	0.88 (0.84-0.91)<br>AIC: 384<br>NfL not significant

AUCs from logistic regression models for predicting Aβ positivity (CSF Aβ42/Aβ40 ≤0.059) in the younger half of the cohort (60-72 years), and the older half of the cohort (73-88 years). Results are shown as areas under the ROC curves (95% CI). AIC shows the model fit in relation to the number of variables (lower = better within the same population). Abbreviations: AIC, Akaike information criterion; AUC, area under the curve; CI, confidence interval; CU, cognitively unimpaired; MCI, mild cognitive impairment.

eTable 7. Demographic and clinical data of the German validation cohort

	CU n=34	MCI n=109	AD dementia n=94	Whole population n=237
**Sex, F/M**	24/10	41/68	55/39	120/117
**Age, years**	59 (11)	65 (9)	70 (9)	66 (10)
**MMSE**	29 (1.4)	27 (1.9)	24 (2.1)	26 (2.5)
**Amyloid positivity [%]**	18	48	80	56
**CSF**				
**Aβ42, pg/mL**	1133 (410)	898 (436)	672 (335)	842 (424)
**T-tau, pg/mL**	230 (113)	277 (166)	365 (159)	305 (164)
**Aβ40, ng/mL**	18.3 (6.7)	16.4 (6.1)	17.9 (6.4)	17.3 (6.3)
**Aβ42/Aβ40**	0.064 (0.016)	0.056 (0.021)	0.040 (0.017)	0.050 (0.021)
**P-tau/Aβ42**	0.021 (0.021)	0.041 (0.054)	0.061 (0.046)	0.046 (0.049)
**Plasma**				
**Aβ42, pg/mL**	30.1 (6.5)	27.3 (6.5)	26.1 (6.5)	27.2 (6.6)
**T-tau, pg/mL**	13.8 (4)	14.2 (4.7)	15.3 (4.5)	14.6 (4.5)
**Aβ40, ng/mL**	0.439 (0.102)	0.415 (0.113)	0.437 (0.106)	0.427 (0.109)
**Aβ42/Aβ40**	0.071 (0.016)	0.068 (0.011)	0.061 (0.012)	0.066 (0.013)

Aβ status was defined based on a CSF Aβ42/Aβ40 cutoff of ≤0.059. Data are shown as mean (SD) unless otherwise specified.

©Palmqvist S et al. *JAMA Neurology*.
